# Long-term exposure of endangered Danube sturgeon (*Acipenser gueldenstaedtii*) to bisphenol A (BPA): growth, behavioral, histological, genotoxic, and hematological evaluation

**DOI:** 10.1007/s11356-024-33168-2

**Published:** 2024-04-15

**Authors:** Mert Minaz, İlker Zeki Kurtoğlu

**Affiliations:** Department of Aquaculture, Faculty of Fisheries, Recep Tayyip Erdoğan University, Rize, Turkey

**Keywords:** Bisphenol A, Fish, LC_50_, Endangered species, Pollution

## Abstract

Danube sturgeon (*Acipenser gueldenstaedtii*) which is identified as endangered species can be exposed to pollutants such as bisphenol A (BPA) that have a disruptive effect on the endocrine system at any time. Starting from this motivation, the current study focused on BPA toxicity in *A. gueldenstaedtii* juvenile individuals and its adverse effects in sub-lethal concentration. The median lethal concentration (LC_50_) of BPA was 5.03 mg/L in 96th hour. In the chronic period, 0.625 mg/L and 1.25 mg/L BPA concentrations were evaluated based on the result of acute study. Accordingly, growth performance was significantly decreased in BPA groups (1.25 mg/L BPA group was significantly lowest) compared to control (*p* < 0.05). In the acute period, behavioral disorders were standing at the bottom/corner of tank, slowing and stopping of gill movement, decreased response to stimuli, and death, respectively. While vacuolization was severe in the liver tissue of the fish in the acute period, intense necrosis and melanomacrophage centers were observed in the chronic period. In terms of genotoxicity, longer DNA migration was observed in all groups exposed to BPA than in the control group. In addition, lower erythrocyte and hemoglobin were observed in the BPA groups compared to control. As a result, the current study revealed toxic effect of BPA on *A. gueldenstaedtii* juvenile individuals and its negative results on fish physiology.

## Introduction

Bisphenol A (BPA) is a synthetic monomer used in the plastics industry that has a negative effect on the endocrine system (Vandenberg et al. 2010; Rubin 2011; Rebai et al. 2021). Especially with the increase in plastic production year by year, BPA accumulation in aquatic environments becomes hazardous. It has been reported that around 1 million pounds of BPA is released into the environment annually (McCracken et al. 2017). The presence of BPA in natural waters has been reported in various studies with both lower and higher concentrations ranging from 4.4 to 8000 ng/L (Belfroid et al. 2002; Jonkers et al. 2010; Lee et al. 2013). Although even the current highest concentration seems low, future projections support that BPA tends to increase rapidly in natural waters depending on plastic consumption (Flint et al. 2012). In addition, residual BPA concentrations have been observed in aquatic organisms, which are indispensable for human consumption as protein food (Belfroid et al. 2002; Basheer et al. 2004; Mita et al. 2011). Therefore, indirect exposure of BPA to humans in the food chain is inevitable. However, BPA production tends to increase continuously due to heavy plastic consumption (Vandenberg et al. 2009; Onay et al. 2023).

BPA has been reported as a toxic/moderately toxic chemical for vertebrates and invertebrates (Minaz et al. 2022b, 2023). In fish exposed to BPA, growth slows (Mccormick et al. 2010), reproduction is negatively affected (Bhandari et al. 2015), and mortality increases (Hanson et al. 2014). BPA disrupts the endocrine system, including acting as an estrogen agonist (Le Fol et al. 2017). BPA is more effective on organisms in the larval period than in the adult period (Brown et al. 2019). Fish are exposed to BPA during the larval period, which is the most vulnerable stage to external stressors (Minaz et al. 2024). However, in order to express this clearly, it needs to be investigated in individual fish species. Exposure of aquatic organisms to toxic substances such as BPA is an important mortality factor. The lower the trophic level of the organism exposed to BPA, the higher the effect of the exposure. For example, while the median lethal concentration (LC_50_) value of BPA is between 0.96 and 2.70 mg/L for aquatic invertebrate organisms, it varies between 6.8 and 17.9 mg/L for fish (Mathieu-Denoncourt et al. 2016). Organisms with low trophic levels have a short life cycle and respond quickly to environmental changes (Ventura et al. 2010).

Exposure to BPA in the aquatic ecosystem can be considered as a potential risk to all organisms (Diler et al. 2022). Especially for critically endangered species such as Danube sturgeon (*Acipenser gueldenstaedtii*) (Gessner et al. 2022; IUCN 2024), it is very important to investigate BPA and similar endocrine-disrupting toxic substances. Because sturgeon has delicious meat and high-value caviar, it is a commercial species for aquaculture and fishing (Song et al. 2022). This calls into question the potential of sturgeon exposed to BPA to be carriers of an endocrine-disrupting chemical onto the dinner table. In addition, the fact that they reach reproductive maturity in a long time and are in danger of extinction makes local governments even more sensitive for sturgeon. Anadromous migratory Danube sturgeon migrates between the Black Sea and the Danube River. They migrate more than 1200 km upriver, especially to spawn (Strat and Gheorghe 2023). It was previously stated that the Danube River, where *A. gueldenstaedtii* is most frequently reported, contains BPA because it is a very long river passing through the borders (city centers) of many countries (Chiriac et al. 2021). Therefore, the physiological response of Danube sturgeon exposed to BPA has become an interesting ecotoxicological issue. In light of all these reasons, a series of studies are needed to monitor the sturgeon, to determine its resistance to toxic substances and to take it under protection. For instance, it has been reported that BPA concentrations found in nature can degrade the antioxidant system in starlet (*Acipenser ruthenus*), thus causing a decrease in sperm quality and DNA damage (Hulak et al. 2013). On the other hand, in the BPA toxicity study conducted on Danube sturgeon larvae, 24th, 48th, and 72nd hour LC_50_ values were 803.4 μg/L, 63.1 μg/L, and 39.6 μg/L, respectively (Minaz et al. 2024). According to our null hypothesis, we consider that the BPA has the potential to have a toxic effect on Danube sturgeon juvenile. Current study reveals the LC_50_ of BPA on *A. gueldenstaedtii* juvenile. Afterwards, adverse effects of sub-lethal BPA concentration prepared based on LC_50_ were examined in the chronic period. The lack of previous studies on the potential effect of BPA, especially on sturgeon juvenile, is an important novelty that will fill this gap in the literature.

## Material and method

### System design and water quality parameters

*A. gueldenstaedtii* juvenile (~ 1 year) were provided from Aquaculture Application and Research Center in Recep Tayyip Erdoğan University. Because the fish have not reached sexual maturity, their gender is uncertain in this stage. Fish were placed in the tanks 1 week before the acute and chronic trial for adaptation. Trials were established in the 50 L aquariums with rested tap water under the condition of manipulated photoperiod (12 h light and 12 h dark). Air stones were used for aeration for each tank. Water exchange was not applied in the acute period since fish were not fed, whereas 100% water was daily changed in the chronic period. All experimental studies were checked and approved by the Ethical Local Committee of the Recep Tayyip Erdogan University (Decision No: 2023/05).

A portable multi-parameter (Hach, HQ40D 58258–00) was daily used for measurement of pH, dissolved oxygen (DO; mg/L), temperature (°C), and electrical conductivity (EC; µS/cm) as water quality parameters. Accordingly, pH (7.18 ± 0.3), DO (7.48 ± 0.4 mg/L), temperature (23.2 ± 0.8 °C), and EC (98.45 ± 12.87 µS/cm) were measured within the limit values that would not adversely affect the fish during experiments. Finally, water samples from each aquarium were analyzed in terms of BPA to prove the presence of BPA-free water.

### Acute and chronic toxicity testing

All experiments in the acute and chronic period were processed in the Aquaculture Application and Research Center and toxicology laboratory at the Faculty of Fisheries, Recep Tayyip Erdoğan University, Rize, Türkiye. BPA (97% purity, Sigma Aldrich) used in chronic and acute trials was purchased commercially. The lethal concentration (LC_50_) that killed 50% of the target population was calculated in 24, 48, 72, and 96 h. Ten fish in triplicate (thirty fish in total) were used for each concentration group (six groups including control) in the experimental design. Five different BPA concentrations (3.0 mg/L, 4.5 mg/L, 6.0 mg/L, 7.5 mg/L, and 9.0 mg/L) and one control (no BPA exposure) were applied under static conditions in the acute period. The LC_50_ values in the 24th, 48th, 72nd, and 96th hour were calculated as 6.84, 6.14, 5.49, and 5.03 mg/L, respectively (Table 1). LC_50_-96 h was used for the measurement of sub-lethal concentration. Accordingly, two different sub-lethal (0.625 mg/L and 1.25 mg/L) BPA concentrations were determined based on 1/8 and 1/4 of the lethal concentration (Abdel-Tawwab and Hamed 2018). Ten fish for each group (thirty fish in total) were placed in triplicate. The chronic trial was established for 28 days under daily renewed water condition (%100 change per day).

**Table 1:** Lethal concentration (LC) of bisphenol A for different exposure periods (24, 48, 72, and 96 h) in *A. gueldenstaedtii*

Concentration (mg/L)	Total fish	Mortality (%)			
		24th hour	48th hour	72nd hour	96th hour
3.0	30	0	0	3	4
4.5	30	0	3	5	6
6.0	30	8	13	16	19
7.5	30	18	23	25	27
9.0	30	30	30	30	30
LC_10_ (mg/L)		5.57	4.65	3.61	3.35
LC_50_ (mg/L)		6.84	6.14	5.49	5.03
LC_90_ (mg/L)		8.4	8.11	8.35	7.84

To monitor BPA concentrations, 20 mL water samples were taken from each tank at the end of the periods in the acute study and weekly in the chronic study. Before analysis, the samples were conditioned with ammonium hydroxide and filtered through 0.2-micron filter papers. It was subsequently analyzed in a negative ionization LC–MS/MS system with a detection limit of 0.2–0.3 µg/L.

### Monitoring of growth performance and behavioral changes

Fish were weighted by precision scales before trial to determine initial weight. Fish were daily fed (2% of the fish biomass) with commercial pelleted diet (~ 35% protein, Skretting). At the end of chronic experiment, fish were collected from each aquarium and specific growth rate (SGR); weight gain ratio (WGR), feed conversion ratio (FCR), and feed efficiency ratio (FER) were calculated as per the following equations:

$${\text{SGR}}\%/d=\left({\text{lnFW}}-{\text{lnIW}}\right)/t\times 100$$

$${\text{WGR}}=\left({\text{FW}}-{\text{IW}}\right)/{\text{FW}}\times 100$$

$${\text{FCR}}={\text{FI}}/{\text{WG}}$$

$${\text{FER}}=1-{\text{FCR}}$$

where FW and IW are the weights of fish in final and initial of trial, respectively. In addition, FI, WG, and *t* represents feed intake, weight gain of fish, and experiment period (28 days), respectively.

Fish behaviors were monitored by the same researcher to avoid observational errors during acute period. Behavioral alterations were noted considering the moment when majority of the population (> 50%) exhibit the same behavior. Accordingly, eight different behaviors were taken into account from normal behavior to first death. It was inspired by another study for determining the behavioral endpoints over time (Kane et al. 2004).

### Histological evaluation

Individuals in the chronic and acute periods were randomly taken for histological analyses. Fish were euthanized by clove oil before sampling. The liver and gill tissues were considered for histological evaluation. The neutral buffer solution (10%) was used for fixation of tissues. In the following day, tissues were placed in ethanol (50%) and stored at room temperature. All tissues were exposed to flowing water to remove ethanol. Then, tissues were treated by alcohol (80%, 90%, and 99.9% ethanol series, respectively “15 min per stage”) and xylene (99.9% throughout 15 min × 2 times) series and exposed to paraffin overnight at + 65 °C before the staining stage. Tissues were submerged and blocked into the paraffin in the last stage of the first step. Afterward, paraffin-blocked tissues were cut with a thickness of 5 µm with a microtome and placed on the slide. Slides were kept at + 65 °C overnight to remove paraffin. In the next stage, the tissues were again treated by alcohol (96% and 99.9% during 1 min) and stained with hematoxylin (5 min) and eosin (5 min) series then completed by xylene (1.5 h × 2 times). In the final, the slides were covered with an Entellan™ and cover-slip. Finally, preparates were examined under a light microscope.

A semi-quantitative model developed by Bernet et al. (1999) was used to evaluate quantitatively histological alterations (Table 2). Based on this model, histological alterations were divided into five reaction patterns (circulatory disturbances, regressive changes, progressive changes, inflammation, and tumor). In the current study, only some of these reaction patterns were observed. One importance factor (from 1 “minimal” to 3 “marked) for each reaction pattern was determined in this model. In addition, a Likert scale (from 0 “no change” to 6 “very severe”) was used to score severity of symptoms. In the final, the evaluation of each symptom was calculated by multiplying the importance factor and the score value. The final score for each pattern is determined by summing the assessment scores of the lesions.

**Table 2:** Histopathological assessment tools for tissues based on model of Bernet et al. (1999). Importance factor (1–3) is composed of the respective organ, the reaction pattern, and the alteration. Score value is a Likert rating scale ranging from 0 to 6

Organ	Reaction pattern	Alteration	Importance factor	Score value	Index
Gill	Circulatory disturbances	Hyperemia	IF_1_ = 1	SV_1_ = 0–6	GI_CD_
	Regressive changes	Architectural, structural alterations	IF_2_ = 1	SV_2_ = 0–6	GI_RC_
		Plasma alterations	IF_3_ = 1	SV_3_ = 0–6	
		Necrosis	IF_4_ = 3	SV_4_ = 0–6	
	Progressive changes	Hypertrophy	IF_5_ = 1	SV_5_ = 0–6	GI_PC_
		Hyperplasia	IF_6_ = 2	SV_6_ = 0–6	
Liver	Regressive changes	Necrosis	IF_7_ = 3	SV_7_ = 0–6	LI_RC_
		Plasma alterations	IF_8_ = 1	SC_8_ = 0–6	
	Inflammation	Activation of the reticuloendothelial system (RES)	IF_9_ = 1	SV_9_ = 0–6	LI_PC_

### Genotoxicity analysis

Comet assay was performed blood samples of fish in the end of both acute and chronic period (Singh et al. 1988). Alkali conditions (pH > 13) were provided to determine erythrocyte DNA damage. Approximately 10 µL blood sampled from caudal vein of fish was treated by phosphate buffer saline (PBS-without Ca^++^ and Mg^++^). Therefore, the cell suspension was formed. Then, 75 µL low melting agarose (LMP-0.5%) was mixed 10 µL cell suspension for final mixed suspension. In the first step, slides were frosted and cover by normal melting point agarose (NMP-1%). Then, slides were covered by this mixed suspension in the second layer. Then, 100 µL LMP agarose was used in the last layer for solidification. Slides were exposed to lysing solution (2.5 M NaCl, 100 mM EDTA, 10 mM Tris, pH 10–10.5, 1% Triton X-100, and 10% dimethyl sulfoxide) overnight at + 4 °C after solidification. After lysing treatment, slides were placed in a horizontal gel electrophoresis and immersed in the fresh alkaline electrophoresis buffer for 30 min at + 4 °C. Thus, the DNA unraveled and converted into single-stranded breaks of alkali-labile zones. Electrophoresis was run at 25 V and 300 mA for 30 min. Subsequently, the slides were washed with neutralized buffer and cold distilled water for 10 min, respectively. In the staining stage, 0.5 µg/mL ethidium bromide was used. Then, slides were washed with distilled water for the prevention of excessive staining. Blood samples in the positive control were treated with 100 µm H_2_O_2_ for 10 min at 4 °C. In the end, slides were examined under the fluorescent microscope (Leica DMR HC, Germany). In addition, cells were analyzed using Comet Score™ 2.0 Software (Tritek Crop., Sumemeruck, VA, USA).

### Hematological indicators

Fish were randomly chosen from aquariums for hematological analyses at the end of acute and chronic periods. After that, fish were anesthetized by clove oil according to Ak et al.’s (2022) procedure. Blood samples were taken from the caudal vein of fish with a 2.5 mL syringe. Then, samples were transferred to EDTA K3 tubes. The erythrocyte (RBC), leukocyte (WBC), hematocrit (HCT), and hemoglobin (HGB) in each blood sample were analyzed by an automatic hematological assay (Prokan6800VET). The automatic hematological assay has been previously calibrated based on the fish and then checked (Minaz et al. 2022b).

### Statistical analysis

All data were presented in mean ± standard deviation. As pre-test, normality test was performed with Kolmogorov–Smirnov test. Based on normality analyses, one-way ANOVA and Kruskal–Wallis *H* test were chosen to determine significant differences between groups for parametric and non-parametric groups, respectively. The significant *p* value was statistically preferred maximum of 0.05. All data sets were analyzed by SPSS 25 software package for Windows (Version 25, IBM Corp., Armonk, New York, USA).

## Results

### Lethal toxicity of BPA to *A. gueldenstaedtii*

**Fig. 1:**
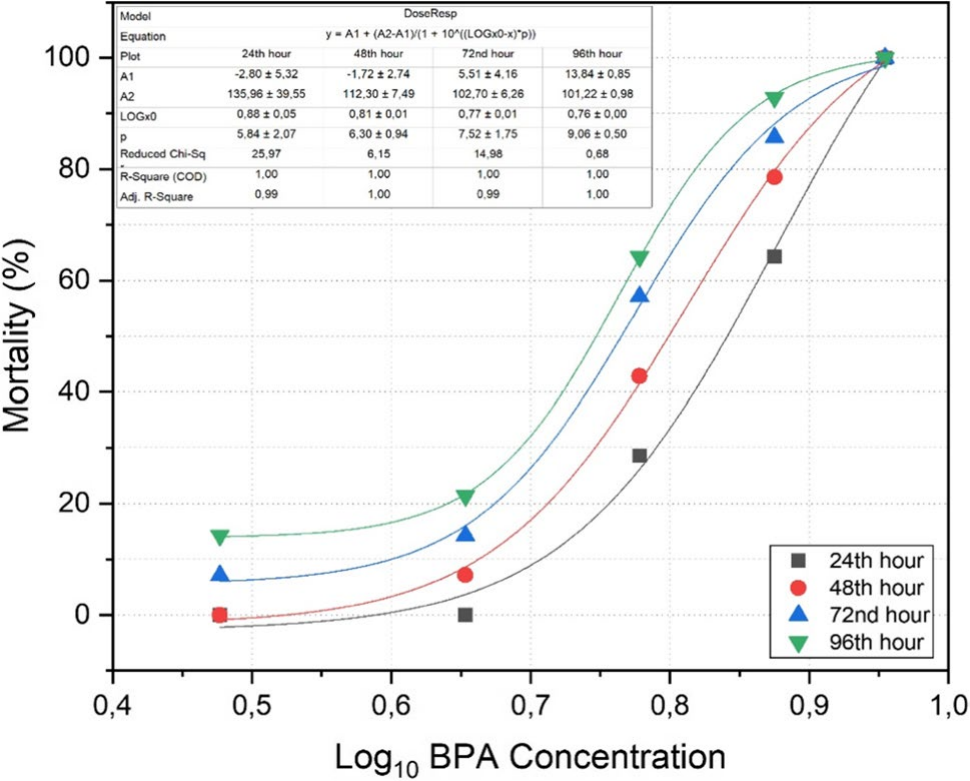
Dose and response graph between Log_10_ BPA concentration and mortality. The mortality rate model is $y=A1+(A2-A1)/(1+{10}^{{{\text{log}}}_{x}0-x}\times p)$. LC_50_ was 5.04 mg/L in 96th h (based on 95% confidence interval)

Figure 1 shows the mortality rate in fish exposed BPA during acute period depending on increased logarithmic BPA concentration. Based on 95% confidence interval, LC_50_ of *A. gueldenstaedtii* was 5.04 mg/L in 96th hour. In addition, mortality rate model [$y=A1+(A2-A1)/(1+{10}^{{{\text{log}}}_{x}0-x}\times p)$] was designed for increased BPA concentration. Variables were assumed according to each exposure period. A1 and A2 are the bottom and top asymptotes, respectively. Moreover, *y*, *x*, and *p* represent mortality rate, BPA concentration, and hill slope, respectively.

### Growth performance and behavioral assessment

The growth performance of fish in control and BPA-exposed groups was observed at the end of the chronic period (Table 3). Accordingly, final weight, specific growth rate, and weight gain rate were significantly higher in 1.25 mg/L BPA exposed group and lower in control group (*p* < 0.01). FCR was significantly higher in control group; 1.25 mg/L BPA-exposed group was significantly higher for FER expressing 1/FCR. Also, no mortality was observed in the groups.

**Table 3:** Growth performance parameters of *A. gueldenstaedtii* after chronic period. IW, initial weight; FW, final weight; SGR, specific growth rate; WGR, weight gain ratio; FCR, feed conversion ratio; FER, feed efficiency ratio

	Control	0.625 mg/L BPA	1.25 mg/L BPA	*F* value	η^2^
IW	51.73 ± 1.1	51.47 ± 0.9	51.17 ± 0.6	2.57	0.461
FW	79.35 ± 1.3^a^	75.96 ± 0.4^b^	69.50 ± 0.7^c^	108.27	0.973
SGR	1.42 ± 0.1^a^	1.29 ± 0.1^b^	1.01 ± 0.1^c^	130.81	0.975
WGR	53.39 ± 2.3^a^	47.58 ± 1.3^b^	35.49 ± 0.9^c^	117.46	0.975
FCR	1.18 ± 0.1^c^	1.31 ± 0.1^b^	1.71 ± 0.1^a^	205.19	0.986
FER	0.85 ± 0.05^a^	0.76 ± 0.01^b^	0.58 ± 0.01^c^	106.20	0.973

^abc^Significant differences between groups for each growth performance indicator (*p* < 0.01)

Fish exposed to different BPA concentrations showed various behavior disorders in the acute period (Table 4). In the highest concentration (9 mg/L), fish showed all behavior disorders into the 12 h, and first death was noted in the end 12nd hour. While no death was observed in the 3 and 4.5 mg/L BPA concentration, first deaths were observed in the 36th and 16th hour for 6 and 7 mg/L BPA concentrations, respectively. There was no weakness in the gill movement and decreased response to stimuli in the minimum BPA concentration (3 mg/L) till end of acute period. The behavioral responses in the current study were designed entirely descriptively, and therefore, there are some limitations in their interpretation and analysis.

**Table 4:** Behavioral monitoring of *A. gueldenstaedtii* in the acute period

	3 mg/L	4.5 mg/L	6 mg/L	7.5 mg/L	9 mg/L
Normal behavior	0 h	0 h	0 h	0 h	0 h
Swimming at the bottom of tank	16–24 h	8–12 h	4–8 h	2–4 h	1–3 h
Standing in the corners	16–24 h	12–14 h	6–8 h	2–4 h	1–3 h
No mobility	24–32 h	18–24 h	8–12 h	4–6 h	4–6 h
Only gill movement	48–72 h	24–36 h	12–16 h	8–12 h	6–8 h
Weakness in the gill movement		36–48 h	16–24 h	10–12 h	8–10 h
Decreased response to stimuli		36–48 h	24–36 h	12–14 h	10–12 h
First death			36 h	16 h	12 h

### Histological alterations

**Fig. 2:**
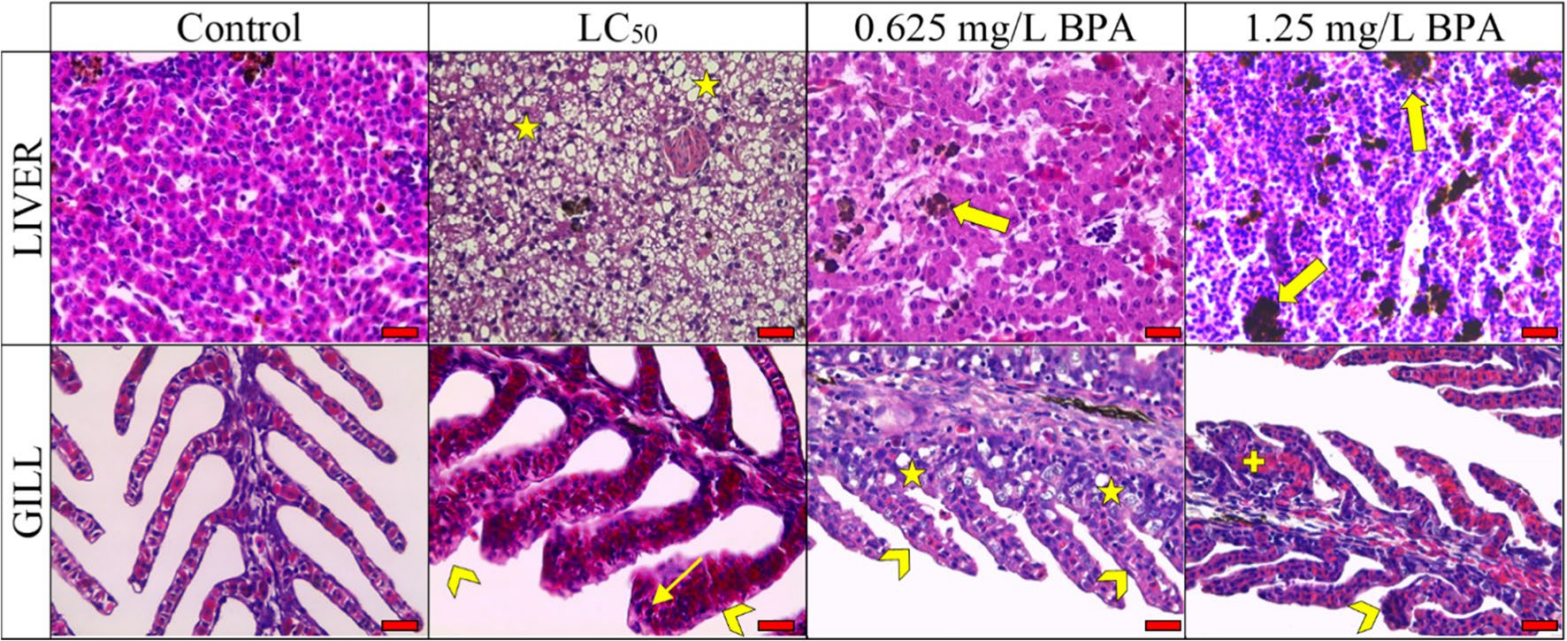
Histological results of liver and gill tissues exposed to BPA in different period. Histological symptoms were marked by various symbols (star: vacuolization, fat arrow: melanomacrophage centers, arrowhead: hyperplasia, thin arrow: hypertrophy, and plus: fusion of seconder lamella). Scale bar 150 µm

Histological analyses were performed in liver and gill tissues (Fig. 2). In the liver tissues of the acute period, very severe vacuolization was observed, while mild necrosis and melanomacrophage centers were formed (Table 5). In addition, the formation of melanomacrophage centers was severe in the chronic period groups. Hyperplasia was dominant symptom in the gill tissues of BPA groups. Moreover, fusion of secondary lamella and necrosis was also severe in the 1.25 mg/L BPA-exposed group. A model was used to determine quantitatively histological alterations (Table 6). The results showed that liver tissues were significantly affected in terms of regressive changes and inflammation in all BPA groups and chronic BPA groups, respectively (*p* < 0.01). On the other hand, reaction indices such as regressive changes, progressive changes, and circulatory disturbances were significantly lower in the control group compared to BPA groups (*p* < 0.01).

**Table 5:** Severity of histological alteration in gill and liver tissues. LC_50_ group is exposed for 96th hour; 0.625 mg/L and 1.25 mg/L BPA groups represent sub-lethal concentration in the chronic period

Tissues	Symptoms	Severities			
		Control	LC_50_	0.625 mg/L BPA	1.25 mg/L BPA
Liver	Vacuolization	-	+ + + +	+	+ +
	Necrosis	-	+	+ +	+ +
	Melanomacrophage centers	+	+	+ + +	+ + +
Gill	Epithelial lifting	-	+	+	+
	Vacuolization	-	+ +	+ + +	+ + +
	Fusion of secondary lamella	-	+ + +	+ +	+ + + +
	Hypertrophy	-	+ +	+	+
	Hyperplasia	-	+ + + +	+ + + +	+ + + +
	Necrosis	+	+ +	+ + +	+ + + +

(-): none, (+): mild, (++): moderate, (+++): severe, (++++): very severe

**Table 6:** The reaction indices of histological alteration during acute and chronic period. CD, circulatory disturbances; RC, regressive changes; PC, progressive changes; CD, circulatory disturbances; IN, inflammation; OI, organ index

		RC	PC	CD	IN	OI
Liver	Control	1.90 ± 2.0^b^	-	-	1.70 ± 0.7^b^	3.6
	LC_50_	8.50 ± 2.0^a^	-	-	0.90 ± 0.7^b^	9.4
	0.625 mg/L BPA	7.60 ± 2.3^a^	-	-	3.80 ± 0.9^a^	11.4
	1.25 mg/L BPA	6.90 ± 2.8^a^	-	-	3.40 ± 1.0^a^	10.3
	*F* or *H* value	16.51^*^	-	-	27.74^**^	
	η^2^	0.645			0.534	
Gill	Control	1.20 ± 1.0^c^	5.10 ± 1.6^d^	0.40 ± 0.5^d^	-	6.7
	LC_50_	3.10 ± 1.0^b^	17.50 ± 3.2^c^	3.30 ± 0.9^b^	-	23.9
	0.625 mg/L BPA	4.40 ± 1.3^ab^	21.10 ± 2.6^b^	1.70 ± 0.7^c^	-	27.2
	1.25 mg/L BPA	5.40 ± 1.1^a^	25.80 ± 3.6^a^	5.00 ± 0.8^a^	-	36.2
	*F* or *H* value	27.34^*^	97.51^*^	33.62^**^	-	
	η^2^	0.714	0.846	0.894		

^*^One-way ANOVA *F* value was presented since data set showed normal distribution. ^**^Kruskal–Wallis *H* values were presented since data set did not show normal distribution. ^abcd^Significant differences between groups for each reaction pattern depending (*p* < 0.01)

### Outputs of DNA damage

**Fig. 3:**
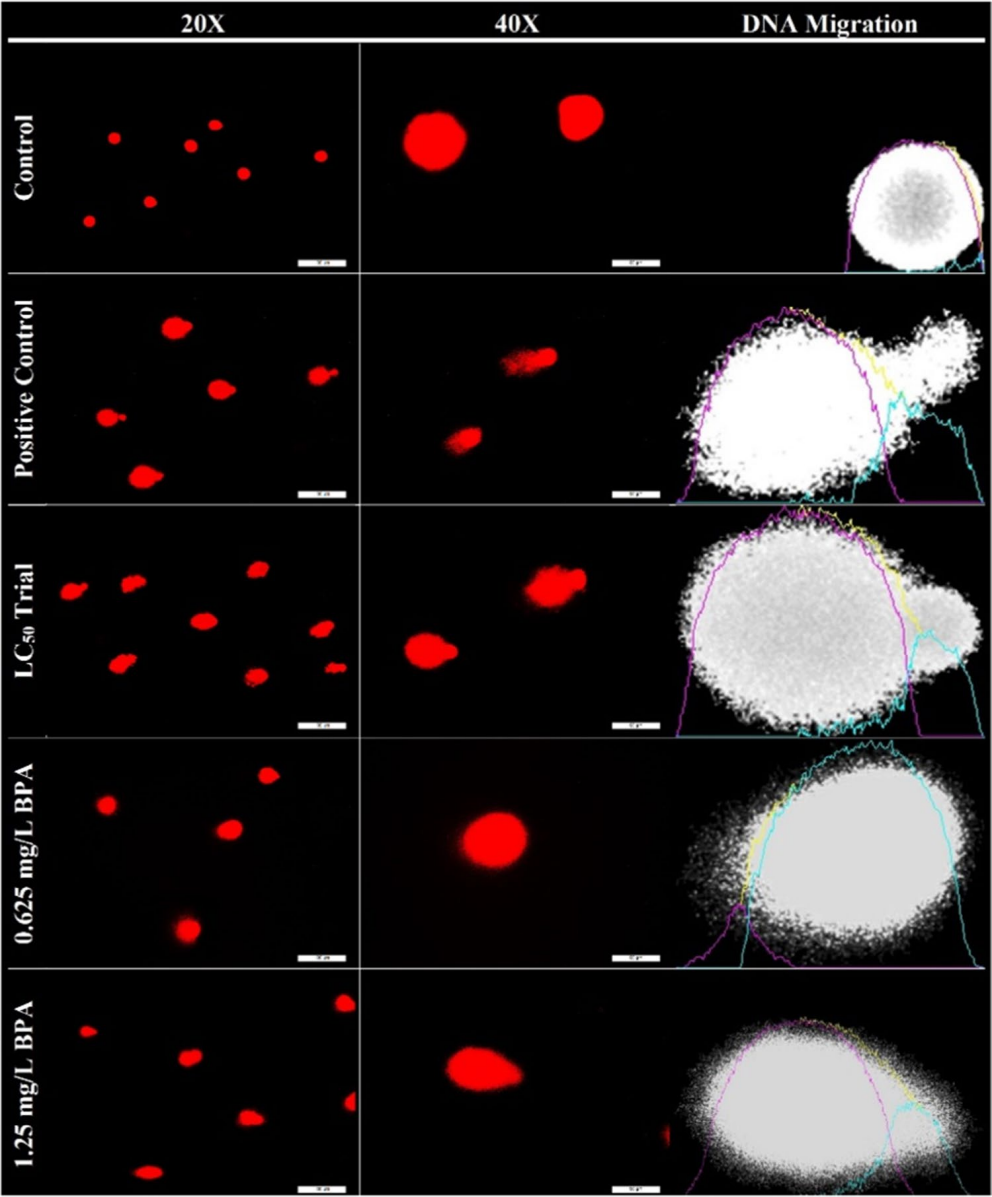
Comet formation in fish erythrocytes for control and experiment group. Positive control represents DNA damaged group by H_2_O_2_. Scale bar 200 µm for 20X and 50 µm for 40X

As a result of alkaline comet assay analysis, DNA migration was observed in the experimental groups exposed to BPA (Fig. 3). DNA damage in the chronic period was affected more than the acute period and control group according to the quantitative index of DNA migration (Table 7). In the control group, head %DNA was significantly highest, whereas tail %DNA, tail olive moment, and tail length were significantly lowest compared to other groups (*p* < 0.01). All parameters were significantly higher in chronic period groups than even positive control and acute period (*p* < 0.01). On the other hand, there were no significant differences between positive control and acute period for tail olive moment and tail length (*p* > 0.05).

**Table 7:** Quantified DNA damage parameters for control and experiment group. Positive control represents DNA damaged group by H_2_O_2_

	Control	Positive control	LC_50_ trial	0.625 mg/L BPA	1.25 mg/L BPA	*F* values	η^2^
%DNA head	95.47 ± 3.1^a^	62.53 ± 8.5^c^	75.55 ± 9.8^b^	37.26 ± 13.9^d^	21.91 ± 11.9^e^	137.38	0.840
%DNA tail	4.57 ± 2.9^e^	37.50 ± 8.7^c^	25.63 ± 12.3^d^	63.53 ± 13.5^b^	78.09 ± 11.9^a^	127.86	0.828
Tail olive moment	1.18 ± 0.9^d^	26.35 ± 6.3^c^	21.83 ± 8.8^c^	47.96 ± 24.2^b^	62.01 ± 16.4^a^	43.76	0.623
Tail length	2.11 ± 2.1^c^	48.96 ± 17.1^b^	56.25 ± 15.5^b^	101.7 ± 49.8^a^	113 ± 28.6^a^	39.78	0.598

### Hematological results

**Fig. 4:**
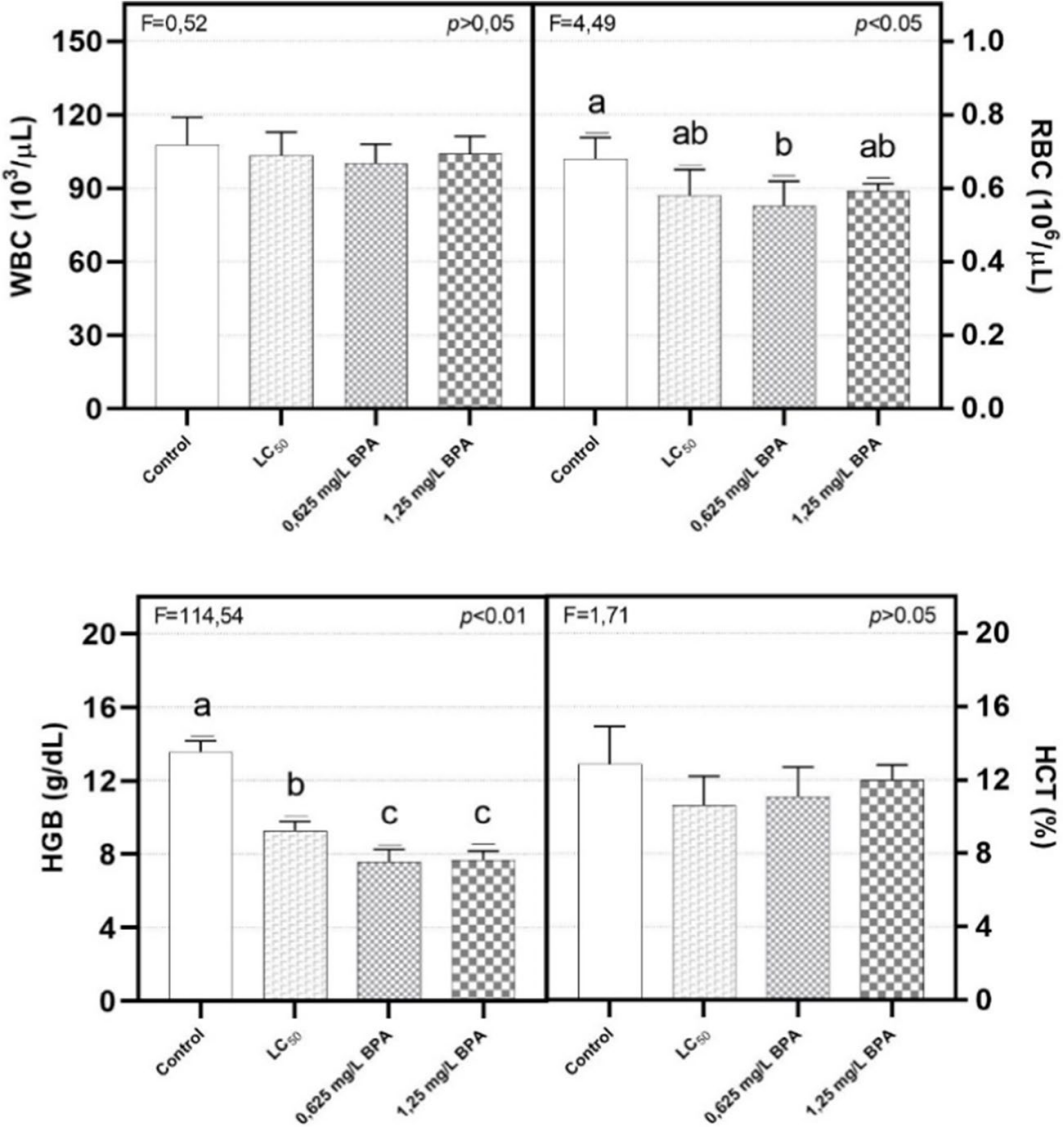
Hematological parameters in *Acipenser gueldenstaedtii* exposed to BPA during different periods. LC_50_ group represents lethal concentration in the acute period (96th h); 0.625 mg/L and 1.25 mg/L BPA groups represent sub-lethal concentration in the chronic period

Figure 4 shows WBC, RBC, HGB, and HCT in different groups as blood indicators. Accordingly, there were no statistically significant differences between groups in WBC and HCT (*p* > 0.05). However, RBC in the control group was significantly higher than 0.625 mg/L BPA-exposed group (*p* < 0.05). On the other hand, HGB in the control group was significantly higher than all BPA-exposed groups, while BPA groups in the chronic period were significantly lowest (*p* < 0.01).

## Discussion

While various pollutants continue to be released into the environment around the world, endangered organisms try to survive and continue their future generations. In this context, countries have many conservation strategies to ensure that endangered animals such as sturgeon can reproduce for future generations (Wang et al. 2011). Even though there are currently strict rules regarding illegal fishing and dams on Danube sturgeon, both negligence in complying with these rules and exposure to toxic substances such as BPA make this fish critically endangered (Gessner et al. 2022). The negative effect of BPA, which is frequently used especially in plastic production, on the endocrine system of aquatic organisms constitutes the motivation of the present study. Although *A. gueldenstaedtii* has a high resistance to conditions such as poor water quality, the fact that this species is in danger of extinction in recent years has brought the possible consequences of possible BPA exposure into the focus of attention. Previous studies have revealed that water quality is adversely affected by acute BPA exposure (Minaz et al. 2022b). In this study, water quality parameters affected the survival standard of *A. gueldenstaedtii* as minimally during the acute and chronic trials. Daily water changes were provided to prevent/minimize possible poor water quality. Thus, it is aimed that all physiological and behavioral changes in fish are due to BPA concentration.

The general physiological response of fish to threatening situations is called stress, and a stress response is initiated immediately upon perception of a stressor (Schreck and Tort 2016). Teleost fish are often used in toxicity studies as they are highly sensitive to toxic substances and present stress responses similar to mammals (Sancho et al. 2000). BPA is classified as “moderately toxic” and “toxic” to the aquatic environment with an LC_50_ value of 1–10 mg/L (Alexander et al. 1988). In various studies of BPA toxicity on fish, the LC_50_ ranged between these values (Faheem and Lone 2013; Asifa and Chitra 2015; Krishnapriya et al. 2017). Based on this issue, five different BPA concentrations were tested in the present study. Especially, 9 mg/L BPA concentration caused the death of all individuals even at the 24th hour. According to the “general adaptation syndrome,” the concept of stress consists of three stages: the “alarm stage,” which activates the body’s defense mechanism, the “resistance stage,” in which tissue damage occurs in the subsequent process, and the “exhaustion stage,” which potentially causes death when the adaptive energy is exhausted (Galhardo and Oliveira 2009). While the alarm reaction and resistance phase of acute stress ended within the first 24 h, high mortality rate was observed with the exhaustion phase in the following process. In addition, the current study will shed light to researchers and commercial fish farming facilities with the dose response model in terms of BPA toxicity. According to this model [$y=A1+(A2-A1)/(1+{10}^{{{\text{log}}}_{x}0-x}\times p)$], the mortality rate in a population exposed known BPA concentration can be estimated. As a result, it is possible for large and small scale facilities to take measures.

Exposure to the toxic material BPA caused an adverse effect on growth performance of *A. gueldenstaedtii* in the current study. Accordingly, final weight, specific growth rate, weight gain rate, and feed efficiency ratio decreased with increasing BPA concentration, while feed conversion ratio increased. Similar negative growth performance in BPA-exposed fish has been observed in previous studies (Chitra and Maiby 2014; Minaz et al. 2022a). Stress and growth axes in fish are intricately interconnected and all stressors, biotic or abiotic, suppress growth performance (McCormick et al. 1998). Against current stress conditions, fish begin to waste/consume energy in a complex manner to restore homeostasis and maintain functional integrity (Guderley and Pörtner 2010). Because the energy available for growth is limited, fish may divert energy substrate away from growth in coping with stress. This negatively affects fish growth performance (Sadoul et al. 2017).

In toxicological studies, the swimming behavior of fish provides an estimate of how the toxic material affects fish welfare. On the other hand, the determination of the lethal concentration is quite effective as a quantitative result. In general, exposure to BPA has previously been reported to produce effects on fish such as loss of mobility and anorexia (Akram et al. 2021a). In the current acute study, the first 24 h can be considered as the critical period. Because it can be characterized as the hour when the inactive waiting (no mobility) starts at a concentration close to LC_50_. This low mobility and poor swimming were observed in our previous study (Minaz et al. 2022b). Moreover, other studies have proven that exposure to BPA causes rapid operculum movement (Naveira et al. 2021; Sharma and Chadha 2021). Different behavioral movements such as jerky movement and erratic swimming have also been observed (Faheem et al. 2017; Akram et al. 2021a). In general, it should not be ignored that the behavioral differences observed in fish exposed to toxic substances may be due to possible neurotoxic effects (Sharma and Chadha 2021). Toxic substances degrade nerve cells, axons, and myelin sheath, altering the synthesis of neurotransmitters involved in behavior (Sharma and Borade 2019).

Histopathological indicators are important biomarkers that reveal population health in an ecosystem (Faria et al. 2014). Histological alterations in the different types of tissues serve as a guide in revealing the biological effect of the toxic substance on fish health and welfare (Rautenberg et al. 2015). In fish, the liver is the main organ for detoxification of xenobiotic pollutants such as BPA (Faheem et al. 2016). Therefore, the liver of fish is an important indicator for estimating the water quality of the aquatic environment (Colin et al. 2016). In this study, severe vacuolization was observed in the acute period, while necrosis and melanomacrophage centers were moderate in the chronic period. Although the presence of melanomacrophage centers in tissues is an expected situation, similar to our study, severe melanomacrophage center is an indicator of a response to toxic substance. Because macrophages are indicator cells of innate immunity in fish and other vertebrates and have the ability to phagocytosis, they have functions such as post-inflammatory repair, tissue regeneration, and elimination of old cells (Sayed and Younes 2017). Vacuolization of hepatocytes is an indication of the disappearance of the cell nucleus and the emergence of the intracellular lipid layer. Therefore, vacuolization can be considered as the primary degradation caused by malathion-like necrosis. Similar necrosis and vacuolization have been previously reported in fish exposed to pesticides (Magar and Shaikh 2013).

Since the gills are the first organs to come into contact with water in fish, the effect of pollutants in the water is primarily evaluated with gill histology (Barišić et al. 2015). The toxic effect of a pollutant is highly correlated with the severity of the damage (Faheem et al. 2016). In the current study, chronic lesions in the gills exposed to 1.25 mg/L BPA concentration were noted. Especially the fusion of secondary lamella has been the dioristic symptom in the chronic period. On the other hand, regressive changes, progressive changes, and circulatory disturbances were significantly detected in the BPA groups compared to the control group. In particular, the main reason of the regressive changes was caused by the intense necrosis symptom in the gill tissues. In our previous study, the necrosis symptom in the gill tissues between the BPA and the control group overlaps with the current study (Minaz et al. 2022a). It has been previously proven that the symptom of necrosis in the gills increases due to the increased BPA concentration (Akram et al. 2021b).

Although fish is an example of model organism to reveal the toxic, carcinogenic, and mutagenic effects of various pollutants in the aquatic ambient (Guerrera et al. 2021), there are limited studies showing that BPA toxicity is genotoxic or mutagenic (Akram et al. 2021a; Frenzilli et al. 2021; Afzal et al. 2022; Ďurovcová et al. 2022). These substances are a source of degradation in the DNA sequence. In this purpose, comet assay technique has been developed to detect DNA damage of organisms exposed to various contaminants (Garaj-Vrhovac and Zeljezic 2000). In contrast to the control group, DNA migration was seen in the groups chronically and acutely exposed to BPA, including the positive control group. Exposure to BPA throughout the chronic period caused significant damage to DNA. Even higher DNA damage was observed than the positive control induced with H_2_O_2_. In a study that revealed the effect of BPA on freshwater with aquatic insect as a bio-indicator species, it proved the disruptive effect of BPA on DNA tail parameters even in the acute process (Martínez-Paz et al. 2013). In a study conducted on rats, it has been proven that BPA has genotoxicity even if it does not provide a mutagenic effect (Tiwari et al. 2012). BPA-induced DNA strand breakage in HepG2 cells in the comet assay (Quinn-Hosey et al. 2012). BPA has been reported to cause DNA strand breaks on aquatic organisms such as *Daphnia magna* and *Chironomus tentans* (Park and Choi 2007).

Hematological alterations are quite rapid tests to quantify stress on fish. Therefore, it can be considered among the primary stress indicators of fish exposed to toxic materials such as BPA. RBC and HGB decreased in the current study depending on BPA exposure. Exposure to sub-lethal BPA concentration during the chronic period resulted in decreased hemoglobin concentration. This reduction in hemoglobin may put a strain on the hematopoietic system of the larvae (Ahmad et al. 2021). The erythropoietic site in fish is the head kidney, and the erythropoiesis process is similar to that in other vertebrates. Fishes show a weak barrier between hematopoietic tissue and circulating blood in which numerous immature cells are present, often comprising over 10% of all erythrocytes. As in other vertebrates, fish erythrocytes contain tetrameric hemoglobin of different oxygen affinity—lower in species living in well-oxygenated water than in those that experience hypoxia (Witeska 2013). In our previous study, the fact that RBC and HGB were lower in BPA-exposed groups was attributed to apoptosis of blood cells by BPA (Minaz et al. 2022a). In relation to histological results, the symptom of hyperplasia and inflammation, especially in the gill tissues, prevented the diffusion of oxygen from the gill surface to the erythrocyte cell membrane (Elahee and Bhagwant 2007).

## Conclusion

The current study focused on the acute and chronic toxicity of bisphenol A in Danube sturgeon (*Acipenser gueldenstaedtii*) juvenile individuals. The lethal concentration of BPA in *A. gueldenstaedtii* was detected after the acute period. The sub-lethal BPA concentration determined according to the acute period affected the fish adversely. The 24th hour was determined as critical point in terms of fish behavior. According to the control group, the growth performance was adversely affected in the experimental group exposed to BPA. In addition, different types of histological symptoms, DNA migrations in erythrocyte cells, and negative results in hematological indicators (red blood cell and hemoglobin) were observed in the BPA groups compared to control. As a result, exposure of endangered sturgeon to BPA in both their natural habitats and aquaculture environments will cause some physiological effects. The current study provides some justification as to why there is a need for more regulation to limit plastic use and waste.

## Data Availability

All datasets used during the current study are available from the corresponding author on reasonable request.
